# Multi-column continuous chromatography effectively improves the robustness of bind-elute mode chromatography in which a pre-elution wash is applied to reduce weakly bound impurities and charge variants

**DOI:** 10.14440/jbm.2025.0105

**Published:** 2025-02-27

**Authors:** Gaoya Yuan, Meng Qu, Yingyue Bu, Xudong Zhang, Yifeng Li

**Affiliations:** Downstream Process Development, WuXi Biologics, Shanghai 200131, China

**Keywords:** Acidic charge variant, Bind-elute mode, Cation exchange chromatography, Multi-column continuous chromatography, Large scale manufacturing, Wash

## Abstract

**Background:**

For the purification of monoclonal antibodies, we employed a wash step in the cation exchange (CEX) chromatography to reduce acidic charge variants. Although optimal wash conditions, determined under a specific loading density, ensured both effective reduction of charge variants and good process yield, applying the same wash conditions to runs where the loading density moderately deviated from the optimal value could result in insufficient reduction of charge variants or low step yield. This issue is particularly problematic with large-scale manufacturing, where the same wash condition (including buffer and volume) is applied across all runs, despite variations in loading density due to fluctuations in harvest titer.

**Objective:**

To address this problem, we intended to demonstrate that multi-column continuous chromatography could offer an effective solution.

**Methods:**

A multi-column setup was implemented, in which all runs except the final one were performed under optimal conditions to ensure both high product quality and yield.

**Results:**

The multi-column approach allowed for both effective charge variant reduction and achievement of good step yield. Although the final run might be conducted under suboptimal conditions, potentially compromising quality or yield, its impact is minimal, as it contributed only a small fraction to the total product, thereby exerting a limited effect on overall quality and yield.

**Conclusion:**

The current study successfully demonstrated the proof-of-concept using CEX chromatography. In fact, the multi-column strategy proposed here may provide a universal solution to the load-dependence issue in wash steps that is aimed at removing or reducing weakly bound impurities or charge variants in any type of bind-elute mode chromatography.

## 1. Introduction

Recombinant monoclonal antibody (mAb) drugs may have charge variants arising from deamidation, oxidation, isomerization, or incomplete C-terminal processing.^1^-^3^ These charge variants are typically classified into acidic and basic species based on their isoelectric points (pIs), with acidic species having lower pIs and basic species having higher pIs than the main species.^4^ The relative abundance of each charge variant can be assessed using capillary isoelectric focusing (cIEF).^4^ To avoid undesired effects, the content of charge variants in certain antibody therapeutics must be maintained below a pre-determined threshold.^5^,^6^ Cation exchange (CEX) chromatography, which separates proteins based on charge, is widely used to separate or reduce charge variants.^7^-^11^

In a recent mAb project, the content of acidic charge variants was required to be kept below 24%. CEX chromatography was employed to meet this specification. Given that acidic species bind more weakly to the CEX column than their main counterpart, a pre-elution wash was introduced to partially remove these acidic variants. This wash must be sufficiently aggressive to reduce the acidic charge variants effectively, yet mild enough to prevent pre-mature elution of the main product. By using a design of experiments (DoE) approach, an optimal wash condition was identified, allowing for the effective reduction of acidic variants without significantly compromising yield. However, the performance of this wash step is highly sensitive to the loading density, and even a moderate deviation from the condition under which the wash was developed can result in either insufficient reduction of acidic variants or significant product loss. This poses a challenge in large-scale manufacturing, where loading densities may vary due to differences in harvest titer, but the same wash conditions (buffer and volume) must be applied across all runs.

In the current work, we demonstrated that multi-column continuous chromatography offered an effective solution to the loading-density dependence on the wash step. A key distinction between single-column batch and multi-column continuous modes is that the latter requires more runs, as smaller columns are used to process the same amount of protein. The fragmentation inherent in the multi-column approach significantly minimizes the impact of deviations in loading density. Although this study focused on CEX chromatography to demonstrate the concept, the multi-column strategy is not limited to this technique. It offers a universal solution to the load-dependence issue associated with wash steps aimed at removing or reducing weakly bound impurities or charge variants in bind-elute chromatography. Beyond improving process robustness, multi-column continuous chromatography also enhances cost-efficiency and productivity.

## 2. Materials and methods

### 2.1. Materials

L-histidine, L-histidine monohydrochloride, sodium acetate trihydrate, sodium chloride, and sodium hydroxide were purchased from Merck (Germany). Acetic acid was bought from J.T. Baker (USA), and sodium phosphate monobasic and sodium phosphate dibasic were procured from Sigma (Germany). BioCore SEC-300 (5 μm, 7.8 × 300 mm) was from NanoChrom (China), and POROS XS resin came from Thermo Fisher Scientific (USA). Fluorocarbon (Fc)-coated cIEF cartridge was purchased from Protein Simple (USA). The mAb used in the current study was expressed in stably-transfected CHO-K1 cells cultured in Hypro 100 culture medium supplemented with Cell Boost 7a and 7b (HyClone). The cells were cultured for 14 days before harvest.

### 2.2. Equipment

Single-column chromatography was performed using an AKTA pure 150, and multi-column chromatography was carried out with AKTA PCC 75, both equipped with Unicorn software version 7.8 (Cytiva, Sweden). pH and conductivity were measured using the SevenExcellence S470 pH/Conductivity Meter (Mettler-Toledo, USA). Protein concentration was quantified on a NanoDrop 2000 spectrophotometer (Thermo Fisher Scientific, USA). Size-exclusion chromatography-high performance liquid chromatography (SEC-HPLC) analysis was conducted by using an Agilent 1260 liquid chromatography system (Agilent Technologies, USA). cIEF analysis was performed by employing an Imaged cIEF Analyzer from Protein Simple (USA). Cell cultivation was carried out in a bioreactor system from Applikon Biotechnology (Netherlands).

### 2.3. CEX chromatography

CEX chromatography was performed using POROS XS resin, by following the protocol outlined in Table 1. The column, with a diameter of 0.66 cm and a bed height of 20.4 cm, had a column volume (CV) of approximately 7.0 mL. To optimize wash conditions, two main factors, *i.e*., histidine concentration (124, 132, and 140 mM) and wash volume (3, 4, or 5 CV), were evaluated using a DoE approach. All runs were conducted at a defined loading density (40 mg of protein per mL of resin). Step yield and acidic species content were the primary responses in the DoE studies. The system was operated at a flow rate that could maintain a 5-min residence time.

**Table 1:** Protocol for cation exchange chromatography

Step	Solution	CV
Sanitization	1 M NaOH	3
EQ	50 mM His-HCl, pH 5.5	3
Load	Neutralized Protein A eluate	/
Wash 1	50 mM His-HCl, pH 5.5	3
Wash 2	124/132/140 (130) mM His-HCl, pH 6.0^a^	3/4/5 (4.8)^a^
Wash 3	60 mM His-HCl, pH 6.0	3
Elution	195 mM His-HCl, pH 6.0	12/5^b^
Strip	50 mM NaAc-HAc, 1 M NaCl, pH 5.5	3
Sanitization	1 M NaOH	3
Storage	0.1 M NaOH	3

Notes:

a Different Wash 2 conditions (histidine concentrations of 124 mM, 132 mM, and 140 mM and wash volumes of 3, 4, and 5 CV) were applied in the wash condition screening studies. Ultimately, 130 mM histidine and 4.8 CV were selected as the Wash 2 condition.

b A12 CV elution was applied in the wash condition screening studies, whereas 5 CV elution was applied in all other studies.

Abbreviations: CV: Column volume; EQ: Equilibration; His-HCl: Histidine hydrochloride; NaAc-HAc: Sodium acetate-acetic acid; NaCl: Sodium chloride; NaOH: Sodium hydroxide.

In the comparison studies, the single-column system used a 1.6 cm diameter column packed with POROS XS resin to a bed height of 21.8 cm (CV: ~43.8 mL). Four cycles were performed at different loading densities (50, 40, 30, and 20 mg/mL). The cycle time for single-column mode lasted approximately 4 h. For the multi-column system, three columns of the same dimension (0.66 cm diameter, 17.7 cm bed height, CV: ~6.1 mL) were packed. Twelve runs (four cycles) were performed continuously without interruption. For all runs except the last one, each column was loaded with 40 mg of protein per mL of resin. The process cycle included the load phase (equilibration, loading, and Wash 1), the wash phase (Wash 2 and Wash 3), and the elution phase (elution, strip, and sanitization). The duration of the load, wash, and elution phases was 44 min, 39 min, and 55 min, respectively. For both systems, the runs were conducted at a flow rate that maintained a 5-min residence time.

### 2.4. Size-exclusion chromatography-high performance liquid chromatography

Size-exclusion chromatography-high performance liquid chromatography analysis was performed on an Agilent 1260 liquid chromatography instrument. For each run, 100 μg of sample was injected into a Nanochrom BioCore SEC-300 stainless steel column (7.8 × 300 mm). The mobile phase consisted of 50 mM sodium phosphate and 300 mM sodium chloride at pH 6.8. Each sample was eluted isocratically for 20 min at a flow rate of 1.0 mL/min. Protein elution was monitored by ultraviolet absorbance at 280 nm.

### 2.5. cIEF

A Protein Simple iCE3 system with an Fc-coated cIEF cartridge was used for this analysis. The master mix contained the following components: 0.5 μL of pI 8.18 marker, 0.5 μL of pI 10.10 marker, 1.0 μL of Pharmalyte 3 – 10, 3.0 μL of Pharmalyte 8 – 10.5, 35.0 μL of 1% methylcellulose, 37.5 μL of 8 M urea, 1.0 μL of 200 mM arginine, and 1.5 μL of ultrapure water. The solution injection was composed of 20 μL of diluted sample at 1.0 mg/mL and 80 μL of master mix. Focusing was performed at 1500 V for 1 min, followed by 3000 V for an additional 8 min.

## 3. Results and discussion

### 3.1. Acidic charge variant reduction by bind-elute mode CEX chromatography

**Figure 1:**
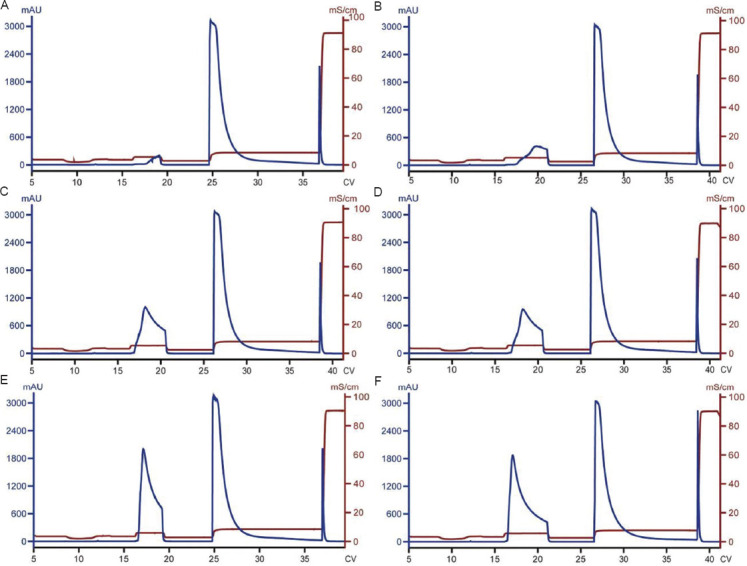
Cation exchange chromatograms of runs conducted under different wash 2 conditions to reduce the content of acidic charge variants. Histidine is the key component of the wash 2 buffer. (A) 124 mM histidine, 3 CV. (B) 124 mM histidine, 5 CV. (C) 132 mM histidine, 4 CV. (D) 132 mM histidine, 4 CV (duplicate of C). (E) 140 mM histidine, 3 CV. (F) 140 mM histidine, 5 CV.

CEX chromatography separates protein species based on charge and is an effective method for controlling charge variant content in mAb purification. In recent work, we utilized CEX chromatography to keep the content of acidic charge variants below 24%, as required (the acidic charge variant content in the CEX load was approximately 29% based on historical cIEF data). The chromatography was conducted under bind-elute mode with a relatively straightforward control strategy. Given that the acidic charge variants bind more weakly than the main species, a pre-elution wash was introduced to partially remove them. While sodium chloride is commonly used for salt gradient elution, preliminary studies suggested that histidine provided better resolution between acidic species and the main species. Therefore, histidine was incorporated into the wash buffer. To identify the optimal wash condition at a defined loading density (40 mg/mL), wash buffers with varying histidine concentrations (124, 132, and 140 mM) were tested. In addition, for each histidine concentration, different wash volumes (3, 4, or 5 CV) were evaluated. As shown in Figure 1 and Table 2, both histidine concentration and wash volume affected the reduction of acidic charge variants and step yield (a larger wash peak in the chromatogram indicates higher product loss, leading to lower yield.). For example, the content of acidic charge variants was reduced to 27.1% and 21.5% under milder wash condition/smaller wash volume (124 mM histidine and 3 CV) and stronger wash condition/larger wash volume (140 mM histidine and 5 CV), respectively (Table 2). Of these two conditions, the acidic charge variant content was reduced to the acceptable level (<24%) only under the stronger wash condition (140 mM histidine and 5 CV). Under these two boundary conditions, the step yield was 88.1% and 56.4%, respectively.

**Table 2:** Yield and quality data of cation exchange eluate from wash condition screening studies

Runs	Histidine concentration (mM)	Wash volume (CV)	Yield (%)	SEC-HPLC (%) HMWs/monomer/LMWs	cIEF (%) Acidic peaks/main peak/basic peaks
Load	/	/	/	2.3/97.7/ND^a^	28.2/63.8/8.0
A	124	3	88.1	1.1/98.9/ND^a^	27.1/66.5/6.4
B	124	5	83.2	1.1/98.9/ND^a^	24.4/68.2/7.4
C	132	4	72.8	1.2/98.8/ND^a^	23.2/68.5/8.3
D^b^	132	4	72.4	1.2/98.8/ND^a^	22.9/69.4/7.7
E	140	3	65.0	1.3/98.7/ND^a^	22.0/69.3/8.7
F	140	5	56.4	1.3/98.7/ND^a^	21.5/69.1/9.4

Notes:

a ND: Not detected;

b Run D was a duplicate of Run C.

Abbreviations: cIEF: Capillary isoelectric focusing; CV: Column volume; HMWs: High molecular weight species; LMW: Low molecular weight species; SEC-HPLC: Size-exclusion chromatography-high performance liquid chromatography.

**Figure 2:**
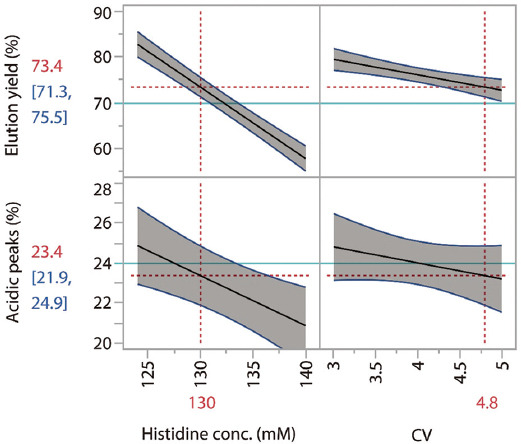
Graphical presentation of data from experiments designed to screen Wash 2 conditions using various histidine concentrations and volumes. These experiments enabled the determination of the optimal wash condition (*i.e*., 130 mM histidine, 4.8 CV) that balances acidic species removal and product yield.

As shown in Figure 2, an analysis of the variance of the CEX data from the aforementioned DoE studies identified the optimal wash condition that balances acidic species removal and product yield. Specifically, 130 mM histidine with a 4.8 CV was selected as the optimal wash condition. Under the selected loading density (40 mg/mL), this wash condition consistently achieved a >6% reduction in acidic charge variants with a step yield exceeding 70%. However, while this wash condition yielded acceptable results, its performance was highly sensitive to the loading density. Lower or higher densities than the selected value (40 mg/mL) resulted in insufficient reduction of acidic charge variants and reduced step yield, respectively (data not shown). This sensitivity is a common issue for wash steps aimed at removing or reducing weakly bound impurities or charge variants in bind-elute mode chromatography. As we have previously noted, the best results (*i.e*., effective impurity removal and good step yield) could only be attained by adjusting the wash conditions according to changes in loading densities.^11^ However, such adjustments are impractical with large-scale manufacturing, where 2–4 cycles of chromatography are typically performed at varied loading densities, but only one wash buffer with a defined composition is prepared in advance and used for all runs. Thus, the load-dependence issue associated with the wash step in bind-elute mode chromatography presents a common challenge in at-scale production.

### 3.2. Multi-column continuous chromatography as a solution to the sensitivity issue of the wash step in bind-elute mode CEX chromatography

To address the sensitivity issue associated with the wash step, we postulated that multi-column continuous chromatography could provide a solution. In a multi-column setup, a greater number of runs are required to process the same amount of loading material compared to single-column batch mode chromatography. As mentioned earlier, for single-column batch mode CEX chromatography, 2 – 4 cycles are typically performed in large-scale manufacturing. Even a single run with a loading density that significantly deviates from the value for which the wash condition was optimized can greatly affect quality or yield. In contrast, the multi-column approach, with a significantly increased number of runs, allows for better control over loading density in all runs except the last one. Although the loading density in the final run may deviate from the preferred value, its impact on overall quality or yield is minimal, as the eluate from this run constitutes only a small portion of the total product. In single-column batch mode, while the claim that loading density can be controlled in all runs except the last one also holds true, the impact of the last run was more significant due to the smaller total number of runs (*i.e*., 2 – 4).

**Figure 3:**
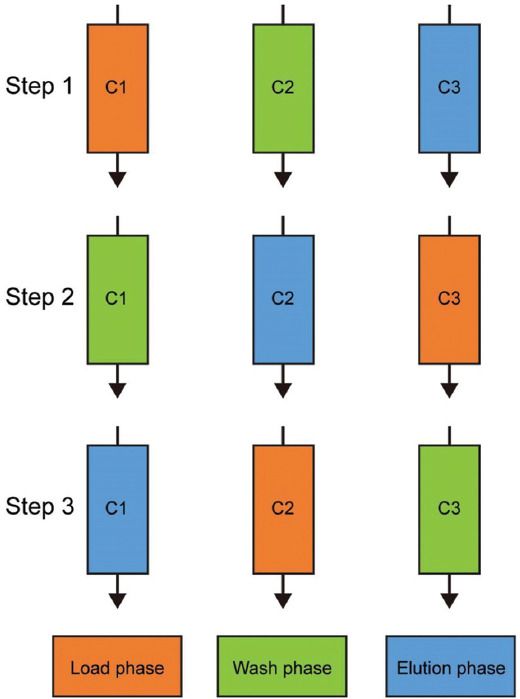
Schematic representation of the stages within a single cycle of the three-column system. C1, C2, and C3 represent columns 1, 2, and 3, respectively. The duration of the load, wash, and elution phases were 44 min, 39 min, and 55 min, respectively. Three columns are the minimum required to synchronize the duration of the different phases.

**Figure 4:**
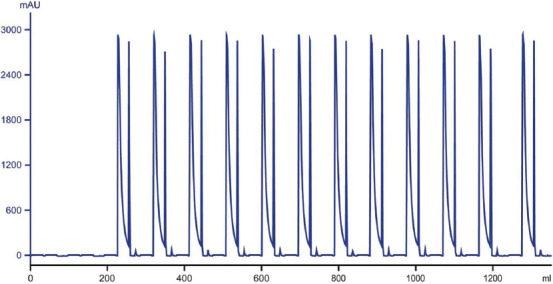
Real-time ultraviolet profiles during continuous sample processing using the three-column system. A total of 12 runs (4 cycles) were performed continuously without interruption, with all runs following the same protocol. For all runs except the last, the column was loaded with 40 mg of protein per mL of resin.

**Figure 5:**
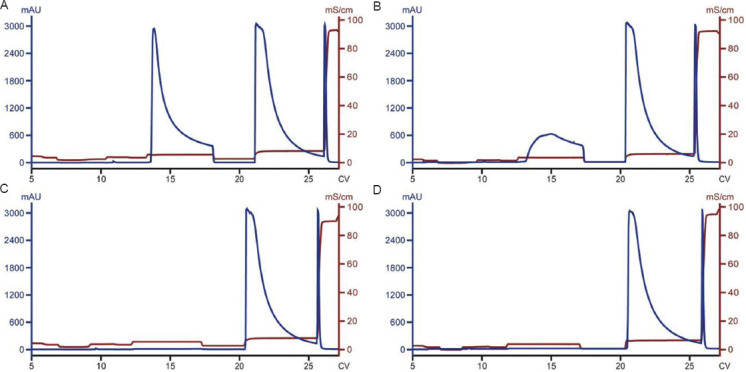
Cation exchange chromatograms of runs conducted at different loading densities. (A) 50 mg/mL. (B) 40 mg/mL. (C) 30 mg/mL. (D) 20 mg/mL. All runs followed the same protocol. At a higher loading density, the wash peak was larger, resulting in compromised yield under the same wash conditions.

To test the effectiveness of the multi-column strategy, we implemented a three-column setup at the laboratory scale, since three columns are the minimum required to synchronize the duration of different phases (Figure 3). In this setup, columns were loaded with 40 mg of protein per mL of resin for all runs except the last one. The previously determined optimal wash condition (*i.e*., 130 mM histidine and 4.8 CV) under the selected loading density was applied to all runs. The performance of the three-column system was then compared to that of the single-column approach (for the three-column and single-column systems, 2.8 g and 6.1 g of protein samples were processed, respectively). As shown in Figure 4, in the three-column setup, 12 runs (4 cycles) were performed continuously without interruption. The chromatograms showed highly consistent elution and strip phases for all runs, indicating stable process performance. Furthermore, as shown in Table 3, the three-column continuous CEX chromatography successfully reduced the content of acidic charge variants from 29.6% to 23.4% and achieved an overall yield of 75.2%. For the traditional single-column approach, four cycles were performed at different loading densities (50, 40, 30, and 20 mg/mL) to simulate variations that could occur in a real-world scenario, and to assess the impact of loading density on process performance under a defined wash condition. The dynamic binding capacity of the CEX resin under the selected condition was approximately 60 mg/mL. However, at the highest loading density (60 mg/mL), poor resolution between the acidic variants and the main species was observed (data not shown). As a result, 50 mg/mL was selected as the maximum loading density for this study. For all runs conducted in the single-column batch mode, the pre-determined wash condition (130 mM histidine and 4.8 CV) was applied, similar to the runs in the three-column system (Figure 5 for corresponding chromatograms). As shown in Table 4, consistent with previous observations, deviations from the 40 mg/mL loading density, under which the optimal wash condition was determined, resulted in poor quality or low yield. For example, at lower loading densities (20 and 30 mg/mL resin), the wash step failed to reduce the acidic charge variants. At the higher loading density (50 mg/mL), the wash step resulted in a 14% reduction in yield compared to the preferred loading density (40 mg/mL).

**Table 3:** Yield and quality data of the cation exchange elution pool generated by the three-column system

Sample	Yield (%)	SEC-HPLC (%) HMWs/monomer/LMWs	cIEF (%) Acidic peaks/main peak/basic peaks
Load	/	2.4/97.5/0.1	29.6/62.8/7.6
Elution pool	75.2	1.2/98.9/ND^a^	23.4/68.7/7.9

Note:

a ND: Not detected.

Abbreviations: cIEF: Capillary isoelectric focusing; HMWs: High molecular weight species; LMWs: Low molecular weight species; SEC-HPLC: Size-exclusion chromatography-high performance liquid chromatography.

**Table 4:** Yield and quality data of cation exchange eluate generated from four runs with different load densities in the single-column system

Sample	Loading density (mg/mL resin)	Yield (%)	SEC-HPLC (%) HMWs/monomer/LMWs	cIEF (%) Acidic peaks/main peak/basic peaks
Load	/	/	2.4/97.5/0.1	29.6/62.8/7.6
Eluate 1	50	61.7	1.3/98.7/ND^a^	24.5/67.0/8.5
Eluate 2	40	76.0	1.0/98.9/ND^a^	23.7/68.3/8.0^b^
Eluate 3	30	91.5	0.7/99.3/ND^a^	31.8/63.0/5.3^c^
Eluate 4	20	88.9	0.4/99.6/0.0	31.6/64.1/4.3^c^

Notes:

a ND: Not detected.

b Under this condition, the corresponding data for the wash are 15.6/83.7/0.6.

c The marginal increase of acidic variants in eluates 3 and 4 was associated with a decrease in basic variants. In fact, both wash and elution performances were influenced by the loading density. At lower loading densities, basic species and aggregates bound tighter than at higher loading densities, leading to better separation when the same elution conditions were applied. This event resulted in a higher percentage of monomers (as indicated by the SEC-HPLC data) and a lower percentage of basic variants in the eluate.

Abbreviations: cIEF: Capillary isoelectric focusing; HMWs: High molecular weight species; LMWs: Low molecular weight species; SEC-HPLC: Size-exclusion chromatography-high performance liquid chromatography.

In addition to the laboratory-scale performance comparison, we conducted a theoretical analysis comparing the single- and three-column setups, assuming 2000 g of protein needs to be processed within 2 days. As shown in Table 5, the three-column approach resulted in a ~75% reduction in resin usage. Compared to the single-column approach, the three-column setup increased productivity by 400%. Therefore, relative to single-column batch chromatography, the three-column continuous chromatography not only enhances process robustness but also improves cost-efficiency and productivity.

**Table 5:** Comparison of three-column continuous mode and single-column batch mode cation exchange chromatography

Items	Batch mode	Continuous mode	Ratio (%)^a^
Column dimension (cm)	30×17.7	10×13.3	NA^b^
Column number	1	3	NA^b^
Resin volume (L)	12.5	3.1	25
Process time (min)	1032	2640^c^	256
Productivity (g/L/day)^d^	80	320	400

Notes:

a Ratio (in percentage) of the number in continuous mode to that in batch mode.

b Not applicable.

c Although the processing time was longer than in batch mode, the process could still be completed within 2 days, as runs were conducted continuously without interruption, similar to batch mode.

d Conductivity was calculated as the amount of protein processed per liter of resin per day.

## 4. Conclusion

In bind-elute mode chromatography, a pre-elution wash is commonly used to remove weakly bound host cell proteins and product-related impurities/variants. For example, we previously showed that a high-salt wash in Protein A chromatography effectively removed half-antibodies, a byproduct associated with asymmetric bispecific antibody production, which binds more weakly than the product.^12^ However, as we have previously noted, loading density significantly impacted the performance of a defined wash step.^12^ To achieve optimal results (*i.e*., effective impurity removal and good step yield), the wash condition (including buffer composition and volume) must be adjusted in line with the loading density. When a fixed wash condition is applied across all runs (as is typical in large-scale manufacturing), deviations in loading density can lead to suboptimal wash performance: either inadequate impurity/variant removal at lower loading densities or significant product loss at higher loading densities.

In a recent project, a wash step was introduced into the CEX chromatography to reduce the content of acidic charge variants to an acceptable level. In large-scale manufacturing, we encountered the same sensitivity issue. As the column size is fixed, variation in harvest titer among batches can result in different loading densities for individual runs. Meanwhile, a wash buffer with a pre-determined composition is prepared in advance and used across all runs. This practice can lead to suboptimal conditions that negatively affect both product quality and yield. To address this issue, we hypothesized that multi-column continuous chromatography could provide a solution. As demonstrated in this study, the multi-column setup allowed all runs, except the last one, to be conducted under optimal conditions, ensuring effective variant removal and good yield. While the final run may be conducted under suboptimal conditions, its contribution to the overall product is minimal, so its impact on quality and yield is limited. The multi-column approach presented here offers a universal solution to bind-elute mode chromatography, where a wash step is employed to remove weakly bound impurities/charge variants, whose performance is sensitive to loading density. In addition, this strategy is applicable in scenarios where impurities or variants bind more tightly than the target molecule, and their removal relies on selective elution conditions that release the target protein while retaining the impurities/variants. In such cases, changes in loading density may cause the pre-defined elution condition to fail, leading to poor impurity removal or low yield. The multi-column approach, which maintains a consistent loading density across virtually all runs, can address this challenge as well. Beyond improving process robustness, the multi-column strategy enhances cost-efficiency and productivity. Finally, the current study serves as a proof-of-concept. In the next phase, we plan to validate this approach at the pilot and Good Manufacturing Practice (GMP) scale. Multi-column capture is already extensively used in GMP manufacturing, and the multi-column approach proposed in the current study is less complex than those used in the capture step, as each column runs independently without the need for column interconnections. Therefore, we do not anticipate significant challenges or concerns at scale-up.

## Data Availability

The data and supporting information are available within the article.
